# Characterization of lncRNA-Driven Networks in Portal Vein Tumor Thrombosis: Implications for Hepatocellular Carcinoma Progression

**DOI:** 10.7150/jca.107270

**Published:** 2025-02-11

**Authors:** Ji Young Kim, So Hee Dho, Lark Kyun Kim

**Affiliations:** Department of Biomedical Sciences, Graduate School of Medical Science, Brain Korea 21 Project, Gangnam Severance Hospital, Yonsei University College of Medicine, Seoul, Republic of Korea.

**Keywords:** Portal vein tumor thrombosis, Hepatocellular carcinoma, Long non-coding RNA, Transcription factors, Gene regulatory networks

## Abstract

**Background**: Portal vein tumor thrombosis (PVTT) is a frequent and serious complication of advanced hepatocellular carcinoma (HCC) that often results in poor prognosis. Although PVTT holds significant clinical relevance, the molecular mechanisms driving its formation are not well understood. Long non-coding RNAs (lncRNAs) have emerged as potential contributors to PVTT progression, prompting this study to explore lncRNAs as potential biomarkers for PVTT.

**Methods**: We analyzed publicly available datasets from the Gene Expression Omnibus to identify differentially expressed lncRNAs and mRNAs across three comparisons: normal vs. HCC, normal vs. PVTT, and HCC vs. PVTT. Transcriptional profiles were characterized, and proteins interacting with HCC- and PVTT-specific lncRNAs were screened using online databases, revealing that all interacting proteins were transcription factors (TFs). We constructed lncRNA-TF-target gene regulatory networks by intersecting TF target genes with differentially expressed genes (DEGs) from each comparison. Protein-protein interaction (PPI) network analysis was performed to identify key clusters and hub genes, with TFs such as AR and ESR1 being highlighted. Gene Ontology analyses were conducted to understand the biological functions of the regulatory networks.

**Results**: The study identified distinct transcriptional profiles for normal, HCC, and PVTT samples. Key regulatory networks, involving lncRNAs, TFs, and target genes, were constructed, and significant hub genes, including AR and ESR1, were identified as potential therapeutic targets. PPI network analysis revealed important clusters associated with PVTT progression, while Gene Ontology analyses provided insights into relevant biological functions.

**Conclusions**: This study presents a novel framework for understanding lncRNA-TF-mediated gene regulation in PVTT. It identifies potential therapeutic targets and prognostic biomarkers that could facilitate the development of targeted therapies for PVTT, offering new opportunities to improve clinical outcomes.

## Background

Hepatocellular carcinoma (HCC) is the most common form of liver cancer, accounting for approximately 80% of all liver cancer cases. Globally, HCC is the sixth most commonly diagnosed cancer and the third leading cause of cancer-related mortality 1. Several risk factors are well-established contributors to the development of HCC, including chronic infection with hepatitis B and C viruses (HBV and HCV), cirrhosis, alcoholic liver disease (ALD), hemochromatosis, and nonalcoholic fatty liver disease (NAFLD) 2. Moreover, the prognosis for HCC is particularly poor when metastasis or vascular invasion occurs 3. One of the most serious complications of advanced HCC is portal vein tumor thrombosis (PVTT), which is observed in 16-30% of patients with HCC 4. PVTT is characterized by the invasive growth of HCC cells within the portal vein and is regarded as a metastatic vascular invasion of advanced HCC 5. PVTT is closely associated with a significantly worse prognosis, making it a critical factor in disease progression and treatment outcomes. Despite efforts to improve survival rates for HCC patients with PVTT, therapeutic options remain limited. While sorafenib, a multi-kinase inhibitor, is approved for advanced HCC, its impact on overall survival in patients with PVTT is modest 6. A deeper understanding of the molecular mechanisms underlying PVTT formation is essential for developing more effective treatments.

In recent years, non-coding RNAs (ncRNAs), which do not encode proteins, have been recognized as key regulators in various cellular processes, including tumorigenesis and cancer progression. Among these, long non-coding RNAs (lncRNAs), defined as ncRNAs longer than 200 nucleotides, have garnered significant attention due to their roles in cancer biology 7. Numerous lncRNAs have been identified as critical regulators of HCC. For instance, HULC (Highly Upregulated in Liver Cancer), the first lncRNA found to be specifically upregulated in HCC, has been shown to promote tumor growth and metastasis 8, 9. Another example, lncRNA-ATB, functions as a competing endogenous RNA (ceRNA), promoting invasion and metastasis by sequestering miR-200 family members, which regulate epithelial-to-mesenchymal transition (EMT) by targeting ZEB1 and ZEB2 10, 11.

While the role of lncRNAs in HCC has been studied extensively, their involvement in PVTT remains less well understood. Some studies have identified recurrently deregulated lncRNAs in PVTT tissues, with potential roles in cell adhesion, immune responses, and metabolic processes 12. For example, HAND2-AS1, a metastasis-associated lncRNA, has been shown to be inversely correlated with DNA methylation at its promoter region in PVTT samples 12. More recently, the TMEM165/hsa-miR-148a/LINC00909 regulatory axis has been identified as a key factor in the development of PVTT in HCC patients 13. However, the specific roles of lncRNAs in PVTT progression are still unclear.

In this study, we aimed to address this gap by analyzing RNA-seq data from the Gene Expression Omnibus (GEO) dataset GSE77509, comprising 60 clinical samples (primary tumor, PVTT, and adjacent normal tissue) from 20 HCC patients. Our analysis provides novel insights into the expression of PVTT-specific lncRNAs and their interactions with regulatory proteins, offering a potential framework for therapeutic interventions and prognostic biomarkers in PVTT.

## Methods

### Dataset acquisition and preprocessing

The RNA-sequencing dataset GSE77509 was obtained from the NCBI GEO database. This dataset includes samples from 19 HCC patients, with each patient providing matched adjacent normal tissues, primary tumor tissues, and PVTT samples. Raw count matrices provided in the dataset were used for downstream analysis. To account for potential batch effects, batch correction was performed using the ComBat function from the sva package in R. This ensured that technical variability across samples did not confound the biological differences between conditions. The batch-corrected data were used for differential expression analysis.

### Differential expression analysis

Differential expression analysis was performed using the DESeq2 package in R to identify both differentially expressed genes (DEGs) and differentially expressed lncRNAs (DE-lncRNAs). Three comparisons were conducted: normal vs. HCC, normal vs. PVTT, and HCC vs. PVTT. The statistical thresholds for each comparison were defined as follows: an adjusted P-value < 0.05 and log2 fold change > 2 for normal vs. HCC and normal vs. PVTT, and a P-value < 0.05 with fold change > 1.5 for HCC vs. PVTT. Genes and lncRNAs with low read counts across samples were filtered out to avoid false-positive results. The normalized count data were analyzed, and lists of DEGs and DE-lncRNAs were generated for each comparison. Volcano plots were generated using the EnhancedVolcano package in R to visualize significantly up- and down-regulated genes and lncRNAs across comparisons.

### Identification of HCC- and PVTT-specific lncRNAs

To distinguish HCC- and PVTT-specific lncRNAs, DE-lncRNAs identified from the three comparisons were intersected and compared. A Venn diagram was generated using the InteractiVenn web tool to identify overlapping and unique lncRNAs across the normal, HCC, and PVTT groups. These specific lncRNAs were selected for further downstream analysis, focusing on their role in disease progression.

### Prediction of lncRNA-interacting proteins and target genes of TF

To explore the potential biological roles of the identified lncRNAs in HCC and PVTT progression, we predicted lncRNA-protein interactions and identified the target genes of transcription factors (TFs) associated with these lncRNAs. Predictions for lncRNA-protein interactions were made using the RNAInter database and only interactions with a confidence score ≥ 0.3 were considered for further analysis. This threshold ensured a high probability of biologically meaningful interactions. In parallel, TF-lncRNA interactions were analyzed using the TRRUST database. This tool identified the target genes regulated by TFs interacting with HCC- and PVTT-specific lncRNAs, providing further insights into the regulatory networks involved in the progression of these conditions 14, 15. The results from RNAInter and TRRUST were subsequently integrated for downstream analysis.

### Gene Ontology (GO) and Kyoto Encyclopedia of Genes and Genomes (KEGG) enrichment analysis

To gain functional insights into the biological roles of DEGs and TF target genes associated with lncRNAs, GO enrichment analysis and KEGG pathway analysis were performed. These analyses were conducted using the clusterProfiler package in R for the complete set of DEGs. The GO analysis categorized DEGs based on their involvement in biological processes (BP), cellular components (CC), and molecular functions (MF), while the KEGG analysis provided insights into the signaling pathways enriched among the identified genes. DAVID analysis was exclusively applied to the TF target genes interacting with HCC- and PVTT-specific lncRNAs to further explore their functional roles. For all enrichment analyses, statistical significance was set at P < 0.05.

### Construction of a protein-protein interaction (PPI) network and identification of hub genes

To further understand the molecular networks associated with HCC and PVTT, a PPI network was constructed for the TF target genes identified in the previous steps. The STRING database was used to predict protein-protein interactions with a confidence score ≥ 0.4, ensuring that only high-confidence interactions were included in the network. The resulting PPI network was visualized using Cytoscape software (version 3.9.1) to facilitate exploration of the molecular interactions 16. Within the PPI network, molecular clusters representing densely connected regions were identified using the MCODE plugin with a node score cutoff of 5 17. These clusters represent potential protein complexes involved in specific biological processes. To identify key regulatory nodes within the PPI network, we applied the Maximal Clique Centrality (MCC) algorithm using the CytoHubba plugin in Cytoscape. This analysis identified the top 10 hub genes with the highest MCC scores. Additionally, subnetworks were extracted from the PPI network to visualize the genes directly interacting with these hub genes.

## Results

### Analysis of DEGs and DE-lncRNAs in HCC and PVTT

**Figure 1:**
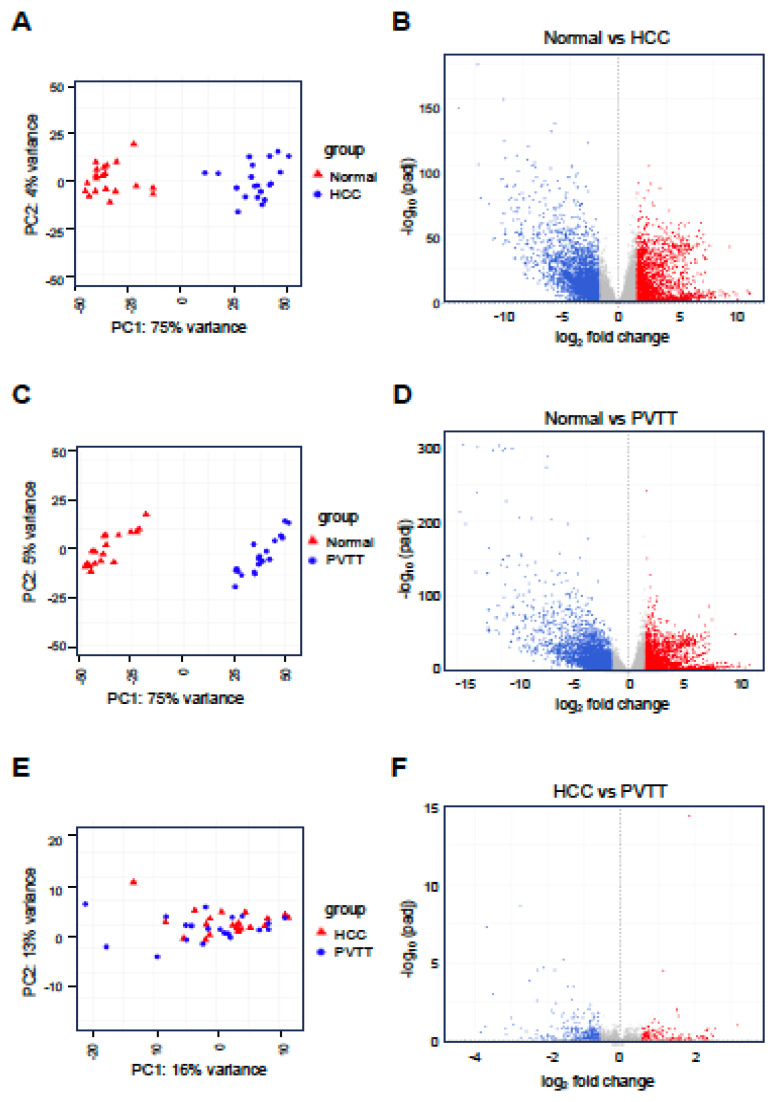
** Analysis of differentially expressed genes (DEGs) in HCC and PVTT.** PCA plot (A, C, and E) for normal tissues (Normal) vs. HCC tissues (HCC) or PVTT tissues (PVTT) and HCC tissues vs. PVTT tissues. Volcano plots (B, D, and F) depict the DEGs, with red and blue dots representing significantly upregulated and downregulated genes, respectively. A threshold of adjusted p-value < 0.05 and |log2 FC| > 1 was set for B and D, while a p-value < 0.05 and |log2 FC| > 0.58 was set for F.

PVTT is a severe complication of advanced HCC characterized by the invasion of tumor cells into the portal vein. This condition significantly complicates treatment and is associated with a poor prognosis. Despite its clinical significance, the molecular mechanisms underlying PVTT formation and progression remain poorly understood. To investigate these mechanisms, we analyzed the expression profiles of lncRNAs and mRNAs in PVTT. We utilized the GSE77509 dataset, which contains RNA-sequencing data from 19 HCC patients, including matched adjacent normal tissues, primary tumor tissues, and PVTT samples. Differential expression analysis was conducted across three comparisons: normal tissue vs. HCC, normal tissue vs. PVTT, and HCC vs. PVTT. The analysis revealed that in the normal vs. HCC comparison, a total of 4,540 genes were differentially expressed, with 1,731 genes upregulated and 2,809 genes downregulated. In the normal vs. PVTT comparison, 5,090 genes were dysregulated, with 1,872 genes upregulated and 3,218 genes downregulated. Furthermore, in the HCC vs. PVTT comparison, 393 genes were differentially expressed, with 117 genes upregulated and 276 genes downregulated (Fig. 1A-F and Fig. S1).

**Figure 2:**
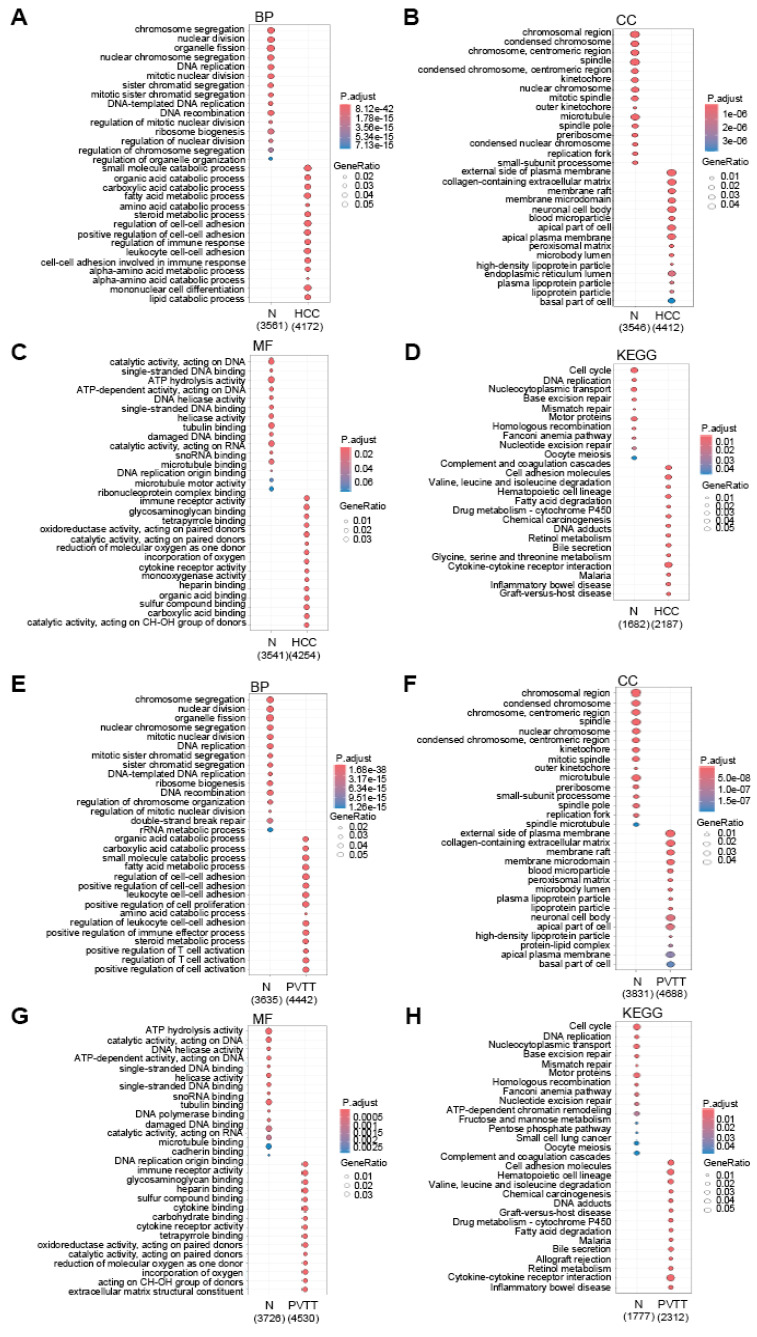
** Gene Ontology analysis of DEGs.** Dot plots showing GO results for DEGs in HCC (A-D) and PVTT (E-H), highlighting GO biological processes (BP) (A and E), cellular components (CC) (B and F), molecular functions (MF) (C and G), and KEGG pathway (D and H). The color intensity reflects the adjusted p-value, while dot size indicates the number of genes involved.

To explore the functional significance of these DEGs, we performed GO functional annotation and pathway analysis (Fig. 2A-H). Interestingly, the GO terms for DEGs in both HCC and PVTT were similar. In the Biological Process (BP) category, DEGs from both HCC and PVTT were enriched in terms related to catabolic processes, cell-cell adhesion, and immune regulation (Fig. 2A and E). In the Cellular Component (CC) category, DEGs were associated with the plasma membrane, lumen, and lipoprotein particles (Fig. 2B and F). In the Molecular Function (MF) category, DEGs were enriched in terms related to immune receptor activity, cytokine receptor activity, and heparin binding (Fig. 2C and G). KEGG pathway analysis revealed that the DEGs were primarily involved in complement and coagulation cascades, cell adhesion molecules, and drug metabolism pathways (Fig. 2D and H).

**Figure 3:**
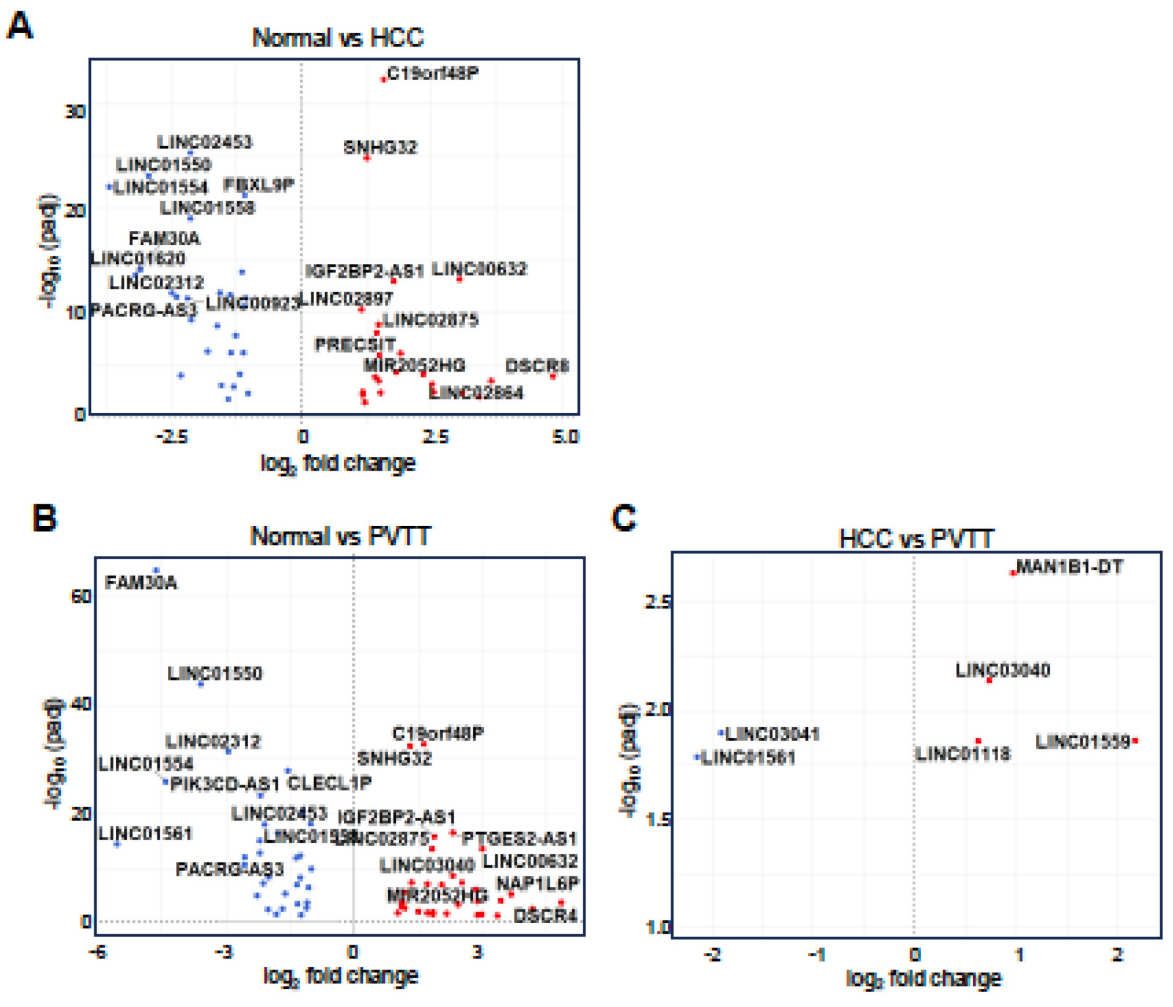
** Analysis of differentially expressed lncRNAs in HCC and PVTT.** Volcano plots depict differentially expressed lncRNAs for normal tissues vs. HCC tissues (A) or PVTT tissues (B) and HCC tissues vs. PVTT tissues (C). A threshold of adjusted p-value < 0.05 and |log2 FC| > 1 was set for A-B, while a p-value < 0.05 and |log2 FC| > 0.58 was set for C.

Given the similarity in GO terms between HCC and PVTT, we shifted our focus to the differential expression of lncRNAs to identify factors that could distinguish PVTT from HCC. As shown in Figure 3, 50 lncRNAs were differentially expressed in the normal vs. HCC comparison, with 23 lncRNAs upregulated and 27 downregulated (Fig. 3A). In the normal vs. PVTT comparison, 64 lncRNAs were dysregulated, with 31 lncRNAs upregulated and 33 downregulated (Fig. 3B). Notably, in the HCC vs. PVTT comparison, only six lncRNAs were differentially expressed, with four upregulated and two downregulated (Fig. 3C).

### Identification of HCC- and PVTT-specific lncRNAs

**Figure 4:**
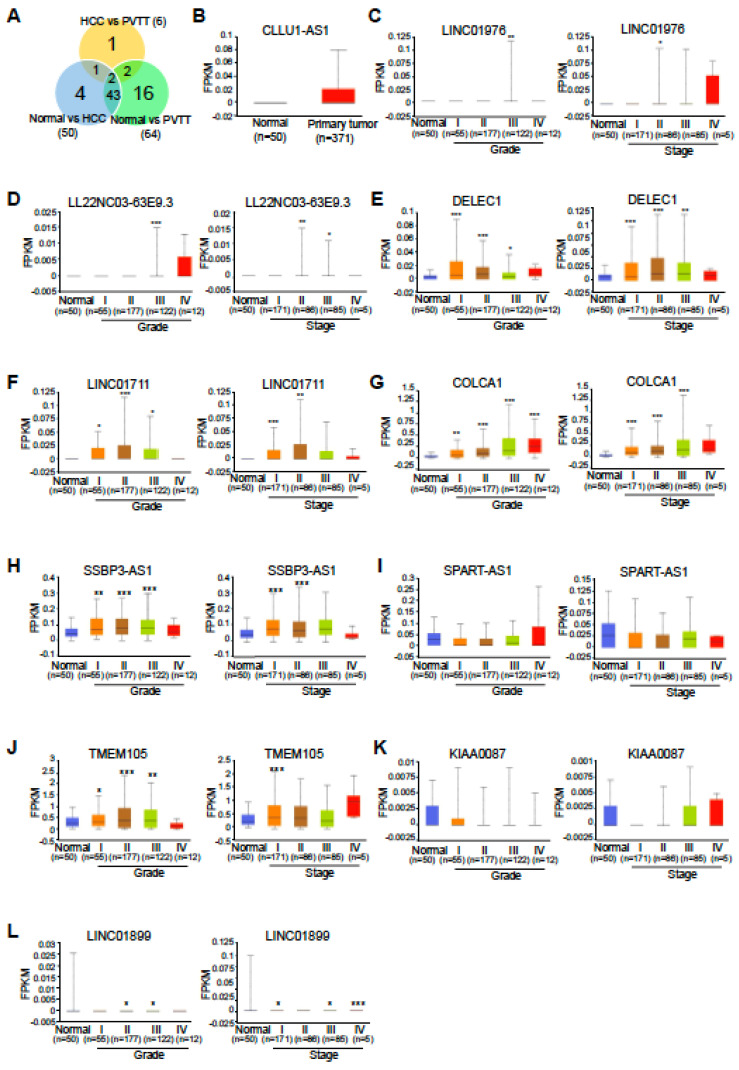
** TCGA lncRNA expression profiles in liver hepatocellular carcinoma.** A. Venn diagram displaying the total numbers of differentially expressed lncRNAs across three comparisons: normal vs. HCC, normal vs. PVTT, and HCC vs. PVTT. B. TCGA expression profiles for CLLU1-AS1 in liver hepatocellular carcinoma samples (n = 371) and normal samples (n = 50). C-L. TCGA lncRNA expression profiles by cancer stage and tumor grade. The number of samples for each tumor grade and cancer stage is indicated in parentheses. *P < 0.05, **P < 0.01, ***P < 0.001.

To further investigate lncRNAs that could account for unique characteristics not shared between HCC and PVTT, we compared lncRNA expression across the datasets. As shown in Figure 4A, a Venn diagram revealed both unique and common lncRNAs among the different groups. Specifically, 20 lncRNAs were found to be unique to the groups (four HCC-specific and 16 PVTT-specific) and these were selected as final targets for further analysis (Table 1).

Next, we assessed the expression levels of these HCC-specific lncRNAs using The Cancer Genome Atlas Liver Hepatocellular Carcinoma (TCGA-LIHC) dataset and examined PVTT-specific lncRNAs in the context of advanced tumor grade and cancer stage from the same dataset, as TCGA-LIHC does not include PVTT samples. Among the four HCC-specific lncRNAs, only CLLU1-AS1 was found to be upregulated in the TCGA-LIHC dataset, while expression data for the other three lncRNAs were unavailable (Fig. 4B). Interestingly, eight PVTT-specific lncRNAs exhibited a gradual upregulation or downregulation in TCGA-LIHC tumor grade and cancer stage, consistent with the RNA-seq data (five upregulated, Fig. 4C-G, and three downregulated, Fig. 4I, K, and L). Additionally, two downregulated HCC-specific lncRNAs were found to be upregulated, which was inconsistent with the RNA-seq data (Fig. 4H and J).

### Identification of lncRNA-interacting proteins specific to HCC and PVTT

**Figure 5:**
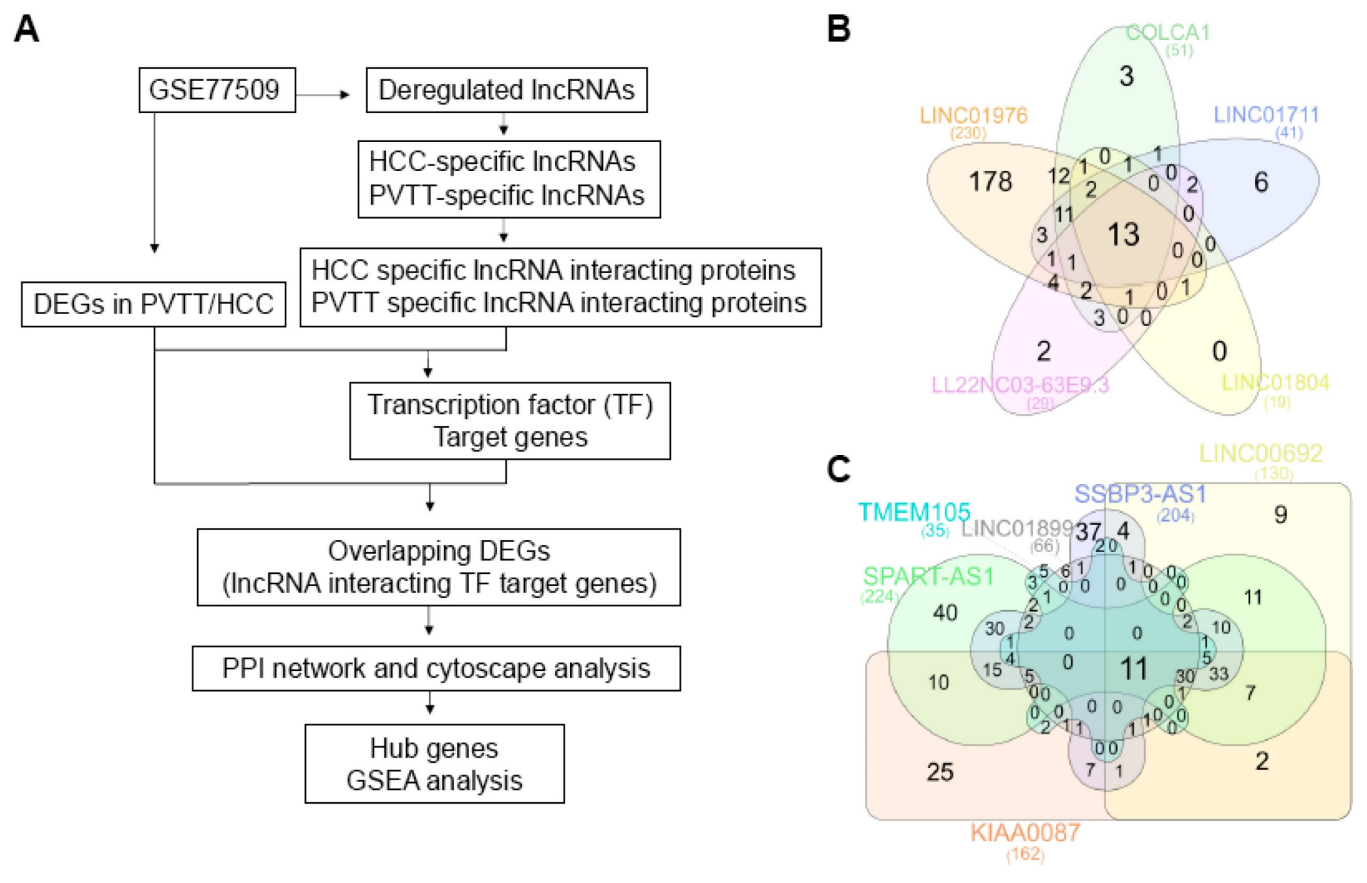
** Identification of PVTT-specific lncRNAs and their interacting proteins predicted by the bioinformatics database, RNAinter.** A. Flowchart outlining the study design. B. Venn diagram displaying the total numbers of differentially expressed lncRNAs across three comparisons: normal vs. HCC, normal vs. PVTT, and HCC vs. PVTT. Venn diagrams illustrating the overlap in PVTT-specific lncRNA interacting proteins among the five upregulated (B) and downregulated (C) groups. Each block represents different types of lncRNAs and the corresponding number of interacting proteins.

Emerging evidence suggests that many lncRNAs function by interacting with specific proteins, playing pivotal roles in various biological processes and diseases. To gain insight into the role of lncRNAs in HCC and PVTT, we explored potential lncRNA-protein interactions using the RNAInter database (Fig. 5A). For the four HCC-specific lncRNAs, we identified 30 proteins that interact with FAM167A-AS1. The other three HCC-specific lncRNAs either had low-confidence interactions or no recorded interactions in the database. To refine the potential targets, we cross-referenced these 30 proteins with the 4,540 DEGs in HCC (normal vs. HCC), which revealed seven overlapping genes: AR, EOMES, ESR1, GATA6, PBX1, SOX2, and TAL1 (Table 2).

For the 16 PVTT-specific lncRNAs, interacting proteins were found for 11 of them. To narrow down these targets further, we divided them into upregulated and downregulated groups. The upregulated lncRNAs had 13 interacting proteins, while the downregulated ones had 11 (Fig. 5B-C). We then compared these interacting proteins with the 5,090 DEGs in PVTT (normal vs. PVTT) and identified four overlapping genes (AR, ESR1, RAD21, and SPI1) for the upregulated group and three overlapping genes (AR, FOS, and SPI1) for the downregulated group (Table 3).

### Functional enrichment analysis of lncRNA-interacting TF target genes

Interestingly, we found that nearly all identified interacting proteins associated with HCC- or PVTT-specific lncRNAs were TFs, with the exception of RAD21. Since TFs play key roles in regulating gene expression, we employed the TRRUST (Transcriptional Regulatory Relationships Unraveled by Sentence-based Text-mining) database to identify the target genes of these TFs. For HCC-specific lncRNA-interacting TFs-AR, EOMES, ESR1, GATA6, PBX1, SOX2, and TAL1-we identified 196 target genes. To refine this list, we cross-referenced these target genes with the 4,540 DEGs in HCC, which yielded 77 overlapping target genes. For the PVTT-specific lncRNA-interacting TFs, we found three overlapping genes (AR, ESR1, and SPI1) in the upregulated group and three (AR, FOS, and SPI1) in the downregulated group. Since AR and SPI1 appeared in both groups, we combined the four TFs (AR, ESR1, FOS, and SPI1) and searched for their target genes using the TRRUST database. After cross-referencing the target genes with the PVTT DEGs, we identified 264 final target genes.

**Figure 6:**
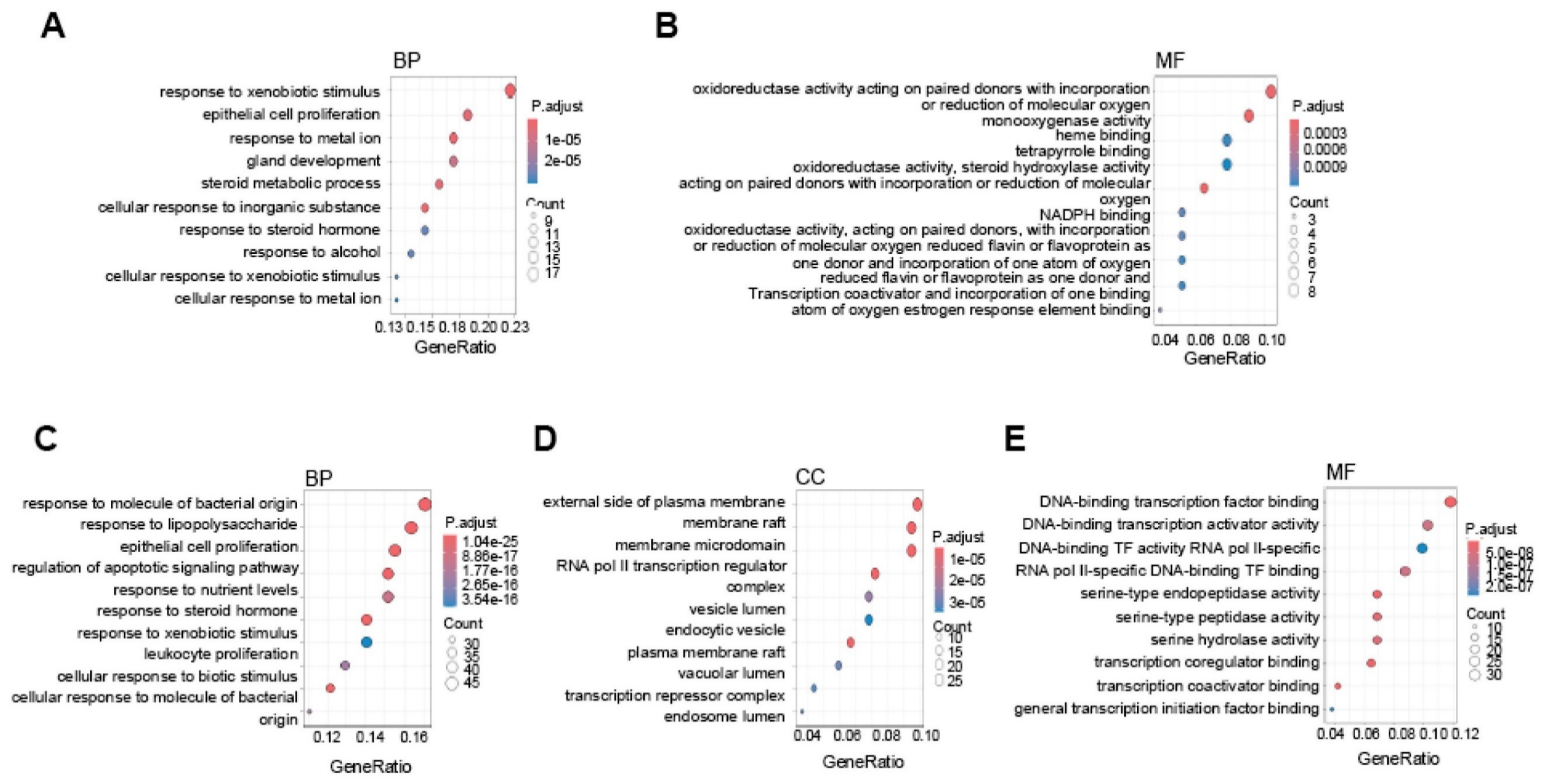
** Gene Ontology analysis of HCC and PVTT-specific lncRNA-interacting transcription factor target genes.** Dot plots showing GO results for HCC-specific upregulated lncRNA-interacting TF target genes, highlighting GO biological processes (A) and cellular components (B). GO results for PVTT-specific lncRNA-interacting TF target genes, illustrating GO biological processes (C), cellular components (D), and molecular functions (E). The color intensity reflects the adjusted p-value, while dot size indicates the number of genes involved.

To further understand the biological significance of these DEGs, we conducted GO analysis. For the HCC-specific lncRNA-interacting TF target genes, the BP category revealed enrichment in processes related to xenobiotic stimulus response, metal ion regulation, and steroid metabolism (Fig. 6A). In the MF category, the genes were associated with oxidoreductase activity, monooxygenase activity, and heme binding (Fig. 6B). In contrast, the PVTT-specific lncRNA-interacting TF target genes were enriched in BP terms related to responses to bacterial molecules and epithelial cell proliferation (Fig. 6C). In the CC category, genes were enriched in membrane and vesicle-related processes (Fig. 6D), while in the MF category, the enriched terms included transcription factor binding, activator activity, and regulator activity (Fig. 6E).

### PPI Network and Hub Gene Identification in HCC and PVTT

**Figure 7:**
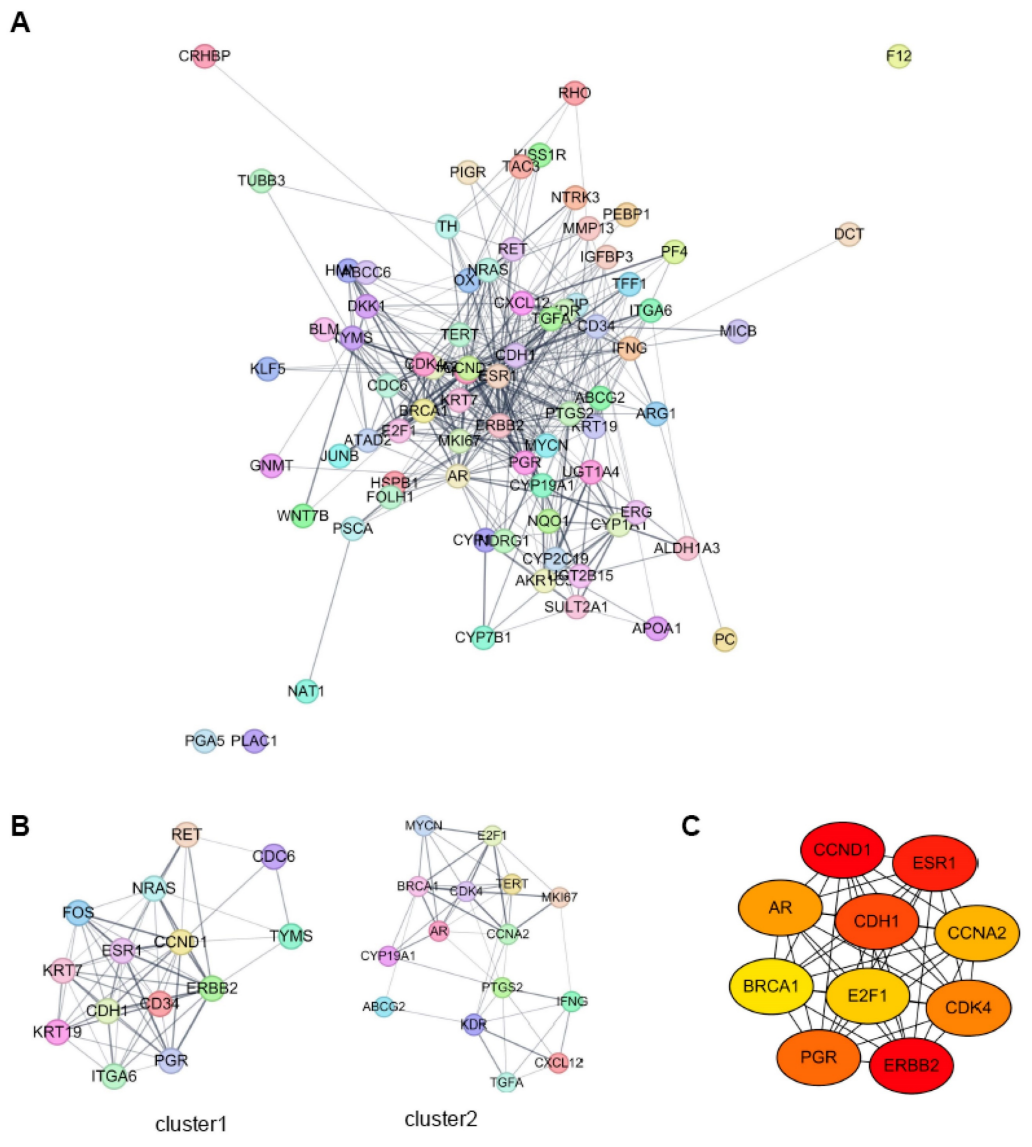
** PPI network analysis of HCC-specific lncRNA-interacting transcription factor target genes using Cytoscape.** A. PPI network analysis of all target genes from the HCC specific lncRNA-interacting TFs. B. MCODE sub-network analysis, highlighting cluster 1 and cluster 2. Cluster 1 contains 14 nodes and 61 edges, while cluster 2 contains 15 nodes and 46 edges. In these networks, nodes represent genes, and edges represent interaction associations between nodes. C. Identification of hub genes within the PPI network. Genes with the top 10 Maximal Clique Centrality (MCC) values are designated as hub genes. Edges represent protein-protein associations, with a color gradient from red to yellow indicating higher MCC rankings of hub genes.

We constructed a protein-protein interaction (PPI) network using the STRING online database and Cytoscape software, incorporating the 77 target genes from HCC-specific lncRNA-interacting TFs (Fig. 7A). Sub-network clustering was analyzed using the MCODE plugin in Cytoscape. Two clusters with a score of ≥5 were identified from the PPI network (Fig. 7B). Cluster 1 comprised 14 nodes and 61 edges, while Cluster 2 contained 15 nodes and 46 edges. Using the Maximal Clique Centrality (MCC) algorithm of the CytoHubba plugin, we selected the ten highest-scoring genes in the PPI network as hub genes. These hub genes were: Androgen Receptor (AR), Breast Cancer 1 (BRCA1), Cadherin 1 (CDH1), Cyclin A2 (CCNA2), Cyclin D1 (CCND1), Cyclin-Dependent Kinase 4 (CDK4), E2F Transcription Factor 1 (E2F1), Erb-B2 Receptor Tyrosine Kinase 2 (ERBB2), Estrogen Receptor 1 (ESR1), and Progesterone Receptor (PGR) (Fig. 7C).

**Figure 8:**
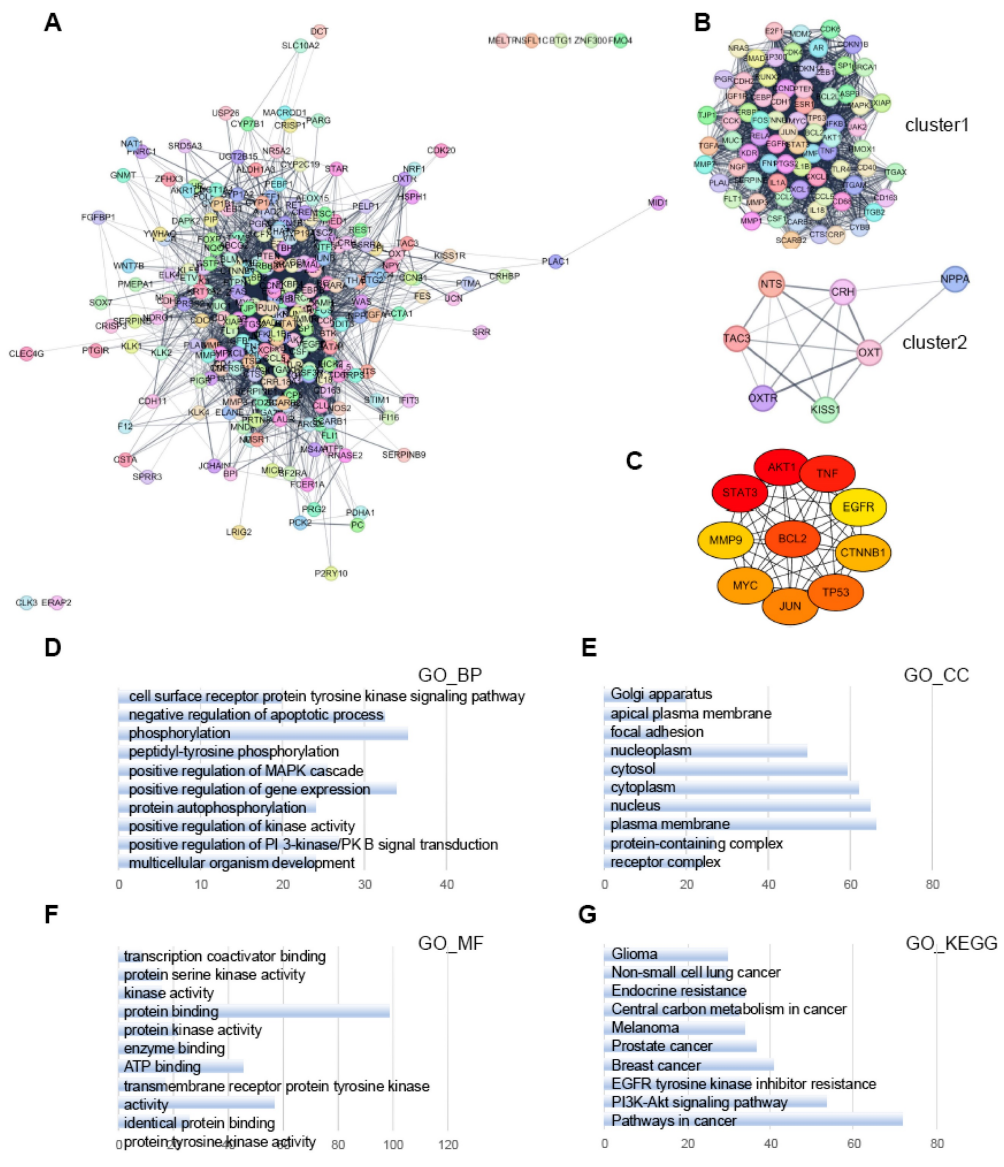
** PPI network analysis of PVTT-specific lncRNA-interacting transcription factor target genes using Cytoscape.** A. PPI network analysis of all target genes from the PVTT specific lncRNA-interacting TFs. B. MCODE sub-network analysis, highlighting cluster 1 and cluster 2. Cluster 1 contains 76 nodes and 1871 edges, while cluster 2 contains 7 nodes and 16 edges. In these networks, nodes represent genes, and edges represent interaction associations between nodes. C. Identification of hub genes within the PPI network. Genes with the top 10 MCC values are designated as hub genes. Edges represent protein-protein associations, with a color gradient from red to yellow indicating higher MCC rankings of hub genes. D-F. GO analysis for genes in cluster 1, focusing on BP, CC, and MF. G. Pathway analysis of cluster 1 genes using the KEGG pathway, conducted with the Database for Annotation, Visualization, and Integrated Discovery (DAVID). The top 10 terms in each category are shown, sorted by gene count.

Next, we created a PPI network using the 264 target genes from PVTT-specific lncRNA-interacting TFs (Fig. 8A). MCODE sub-network clustering analysis identified three clusters with a score of ≥5 from the PPI network; two of these clusters are displayed in Figure 8B. Cluster 1 contained 76 nodes and 1,871 edges, while Cluster 2 had 7 nodes and 16 edges. Using the MCC algorithm of CytoHubba, we identified the ten highest-scoring genes in the PPI network as hub genes. These genes were: AKT Serine/Threonine Kinase 1 (AKT1), BCL2 Apoptosis Regulator (BCL2), Catenin Beta 1 (CTNNB1), Epidermal Growth Factor Receptor (EGFR), JUN Proto-Oncogene, AP-1 Transcription Factor Subunit (JUN), Matrix Metallopeptidase 9 (MMP9), MYC Proto-Oncogene, bHLH Transcription Factor (MYC), Signal Transducer and Activator of Transcription 3 (STAT3), Tumor Protein P53 (TP53), and Tumor Necrosis Factor (TNF) (Fig. 8C).

To investigate the functional significance of the 76 genes in Cluster 1, we performed GO and KEGG pathway enrichment analyses using the Database for Annotation, Visualization and Integrated Discovery (DAVID) (Fig. 8D-G). In the BP category, the Cluster 1 genes were enriched in processes related to the negative regulation of apoptosis, phosphorylation, and positive regulation of gene expression (Fig. 8D). In the CC category, the genes were enriched in annotations related to the cytoplasm and plasma membrane (Fig. 8E). In the MF category, the genes were primarily involved in protein binding and kinase activity (Fig. 8F). KEGG pathway analysis revealed that the Cluster 1 genes were predominantly enriched in cancer pathways and the PI3K-Akt signaling pathway (Fig. 8G).

## Discussion

This study constructs a PVTT-specific lncRNA-TF-target gene network to elucidate the mechanisms underlying PVTT progression. By analyzing the publicly available GEO dataset GSE77509, we identified differentially expressed lncRNAs in HCC and PVTT, distinguishing both shared and unique lncRNAs in these conditions. Through the construction of PPI networks, we further explored the interactions of these lncRNAs with TFs and identified hub genes critical to PVTT development.

Previous studies have shown that PVTT arises from primary HCC nodules via metastatic progression. For instance, Ye *et al.* demonstrated that the gene expression profiles of primary HCC tumors with metastatic potential closely resemble those of their metastases, implying that metastasis-promoting genes are activated within primary tumors 18. Similarly, Wang *et al.* reported that PVTT could form in mice following the inoculation of PVTT-derived HCC cells, exhibiting typical migratory behavior through portal vein branches​ 19. Consistent with these findings, our GO analysis revealed similarities in biological processes enriched in both HCC and PVTT, suggesting that PVTT progression may be driven by factors beyond genetic mutations. This observation led us to focus on lncRNAs as key contributors.

However, our study has limitations. First, the reliance on public datasets without experimental validation limits the originality and applicability of our findings. A critical challenge was the difficulty in obtaining PVTT patient samples and cell lines, which precluded biological experiments that could validate our findings. Nonetheless, given the scarcity of PVTT samples and datasets, analyzing lncRNAs using pre-existing datasets remains highly meaningful, as it provides an initial understanding of the molecular mechanisms underlying PVTT progression. Future studies should address this limitation by acquiring appropriate clinical samples to enable laboratory-based validations, such as qRT-PCR or RNA sequencing, to confirm the expression of PVTT-specific lncRNAs. Additionally, the sample size of 19 patients lacks the statistical power required for conclusive statements. Expanding the cohort size across multiple centers will enhance the robustness of the results. The absence of detailed patient inclusion and exclusion criteria raises concerns about confounding variables, such as coexisting conditions that may influence lncRNA expression. Future research must ensure thorough documentation of clinical parameters to correlate lncRNA profiles with specific patient characteristics and outcomes. Furthermore, we acknowledge the lack of comprehensive clinical data, such as tumor stage and liver function, which would have enabled more precise associations between lncRNA profiles and disease progression. Another limitation is the absence of internal or external validation of the downloaded data. Techniques such as cross-validation with independent datasets for lncRNA-TF interactions will improve the reliability of these findings. To address this, we assessed the expression of HCC- and PVTT-specific lncRNAs using the TCGA-LIHC dataset, which, although lacking PVTT-specific samples, provided insights into lncRNA behavior across different tumor grades and stages. Notably, several PVTT-specific lncRNAs displayed consistent expression patterns across tumor grades, aligning with our RNA-seq data, although some HCC-specific lncRNAs showed inconsistent trends.

Despite these limitations, our dataset analysis revealed several novel lncRNAs specific to HCC and PVTT. Notably, the HCC-specific lncRNA FAM167A-AS1 has been recently linked to gastric cancer progression. The PVTT-specific lncRNA LINC01976, identified as an m7G-related lncRNA, has been implicated in the prognosis of thyroid carcinoma​ 20. Additionally, LINC01711, another PVTT-specific lncRNA, has been shown to play roles in various cancers, including esophageal and renal carcinomas, and promotes hepatic fibrosis by regulating XYLT1, highlighting its importance in liver-related pathologies​ 21. In addition to these lncRNAs, COLCA1 induces oxidative stress in human coronary artery endothelial cells and impairs wound healing​ 22. Similarly, C10orf55 has been identified as a potential biomarker for assessing radiation therapy response in head and neck cancers​ 23. A lactate-responsive lncRNA, TMEM105, promotes liver metastasis in breast cancer by upregulating LDHA and sponging miR-1208 24. Additionally, the downregulation of KIAA0087 enhances osteosarcoma progression by targeting the miR-411-mediated SOCS1/JAK2/STAT3 pathway​ 25.

Notably, we identified six differentially expressed lncRNAs in the HCC vs. PVTT comparison, with four upregulated and two downregulated (Fig. 3C). LINC01559 has been shown to promote tumorigenesis in various cancers, including HCC, where it enhances proliferation by sponging miR-6783-3p, suggesting its role as a potential oncogene 26. Another overexpressed lncRNA, LINC01118, functions as an oncogene and modulates paclitaxel sensitivity in epithelial ovarian cancer by regulating the miR-134/ABCC1 pathway 27. Additionally, SOX2-induced LINC01561 promotes metastasis in non-small-cell lung carcinoma by acting as a ceRNA for SHCBP1 through miR-760 sponging, and is also implicated in breast cancer progression 28, 29. These findings suggest that the lncRNAs differentially expressed between HCC and PVTT may play crucial roles in the progression from HCC to PVTT.

LncRNAs participate in the regulation of various pathophysiological processes through interactions with DNA, RNA, and proteins, including TFs. Our investigation of lncRNA-protein interactions revealed that nearly all PVTT- and HCC-specific lncRNAs interacted with TFs, suggesting that these lncRNAs regulate transcriptional networks essential for tumor progression. For example, PVT1 stabilizes c-Myc expression, promoting oncogenesis​, and PANDA interacts with NF-YA to inhibit pro-apoptotic gene expression under DNA damage conditions 30, 31. Similarly, MEG3 functions as a tumor suppressor by interacting with p53 to activate downstream target genes 32. In our analysis, we identified key TFs such as AR, ESR1, and SPI1 as PVTT-specific, while TAL1, SOX2, and others were exclusive to HCC. GO analysis of TF target genes highlighted divergent biological functions between PVTT and HCC. For example, HCC-specific TF target genes were enriched in oxidoreductase activity and heme binding, whereas PVTT-specific targets were associated with transcription factor regulation and binding activity. These functional distinctions suggest that while HCC-specific lncRNAs may influence metabolic processes, PVTT-specific lncRNAs might play a greater role in transcriptional regulation.

Our study successfully identified distinct lncRNAs and their associated transcription factor networks specific to PVTT in HCC patients. By analyzing GSE77509 dataset, we distinguished both shared and unique lncRNAs in HCC and PVTT, providing novel insights into the molecular mechanisms underlying PVTT progression. The construction of PVTT-specific lncRNA-TF-target gene regulatory networks highlights the pivotal role of lncRNAs in transcriptional regulation associated with metastasis. Furthermore, the identification of key hub genes and transcription factors, such as AR and ESR1, offers potential therapeutic targets and prognostic biomarkers for PVTT. These findings enhance our understanding of PVTT pathogenesis and may inform the development of targeted therapies to improve patient outcomes. Future directions include expanding cohort size, validating findings through experimental approaches, and integrating comprehensive clinical datasets to develop targeted therapies and prognostic biomarkers for PVTT.

## Supplementary Material

Supplementary figure.

## Figures and Tables

**Table 1:** List of overlapping lncRNAs in each analysis set

Differential analysis set	lncRNA	Log2FC
Normal vs. HCC	CLLU1-AS1	2.54
	ARNT2-DT	1.21
	PRSS46P	-1.03
	FAM167A-AS1	-1.41
Normal vs. PVTT	LINC01976	3.37
	LL22NC03-63E9.3	3.00
	LINC01711	2.17
	LINC01804	1.85
	DELEC1	1.71
	COLCA1	1.35
	C10orf55	1.14
	NDUFV1-DT	1.02
	SSBP3-AS1	-1.01
	TMEM14EP	-1.02
	TMEM105	-1.11
	SPART-AS1	-1.24
	LINC02908	-1.26
	KIAA0087	-1.33
	LINC00692	-1.83
	LINC01899	-2.02
HCC vs. PVTT	LINC03041	-1.91

**Table 2:** List of HCC-specific FAM167A-AS1 interacting proteins

lncRNA	Protein Names	description
FAM167A-AS1	AR	Transcription factor
	EOMES	Transcription factor
	ESR1	Transcription factor
	GATA6	Transcription factor
	PBX1	Transcription factor
	SOX2	Transcription factor
	TAL1	Transcription factor

**Table 3:** List of PVTT-specific lncRNA interacting proteins

Overlapping Proteins	Protein Names	description
Upregulated lncRNA-interacting Proteins	AR	Transcription factor
	ESR1	Transcription factor
	RAD21	Cohesin Protein
	SPI1	Transcription factor
downregulated lncRNA-interacting Proteins	AR	Transcription factor
	FOS	Transcription factor
	SPI1	Transcription factor

## Abbreviations

PVTT: portal vein tumor thrombosis
HCC: hepatocellular carcinoma
lncRNA: long non-coding RNA
TF: transcription factor
DEG: differentially expressed gene
GEO: Gene Expression Omnibus
TCGA-LIHC: The Cancer Genome Atlas Liver Hepatocellular Carcinoma
PPI: protein-protein interaction
GO: Gene Ontology
CC: cellular components
MF: molecular functions
KEGG: Kyoto Encyclopedia of Genes and Genomes
MCODE: Molecular Complex Detection
MCC: Maximal Clique Centrality
RNA-seq: RNA sequencing
DAVID: Database for Annotation, Visualization, and Integrated Discovery
TRRUST: Transcriptional Regulatory Relationships Unravelled by Sentence-based Text-mining
STRING: Search Tool for the Retrieval of Interacting Genes/Proteins
HBV: hepatitis B virus
HCV: hepatitis C virus
ALD: alcoholic liver disease
NAFLD: nonalcoholic fatty liver disease
ncRNAs: non-coding RNAs
HULC: Highly Upregulated in Liver Cancer
ceRNA: competing endogenous RNA
DE-lncRNAs: differentially expressed lncRNAs
AR: Androgen Receptor
BRCA1: Breast Cancer 1
CDH1: Cadherin 1
CCNA2: Cyclin A2
CCND1: Cyclin D1
CDK4: Cyclin Dependent Kinase 4
E2F1: E2F Transcription Factor 1
ERBB2: Erb-B2 Receptor Tyrosine Kinase 2
ESR1: Estrogen Receptor 1
PGR: Progesterone Receptor
AKT1: AKT Serine/Threonine Kinase 1
BCL2: BCL2 Apoptosis Regulator
CTNNB1: Catenin Beta 1
EGFR: Epidermal Growth Factor Receptor
JUN: JUN Proto-Oncogene, AP-1 Transcription Factor Subunit
MMP9: Matrix Metallopeptidase 9
MYC: MYC Proto-Oncogene, BHLH Transcription Factor
STAT3: Signal Transducer and Activator of Transcription 3
TP53: Tumor Protein P53
TNF: Tumor Necrosis Factor
