# Supplementary figures and images for: Characterization of lncRNA-Driven Networks in Portal Vein Tumor Thrombosis: Implications for Hepatocellular Carcinoma Progression
Source: J Cancer. 2025 Feb 11;16(6):1754–67. doi: 10.7150/jca.107270 (PMC11905401; doi:10.7150/jca.107270)

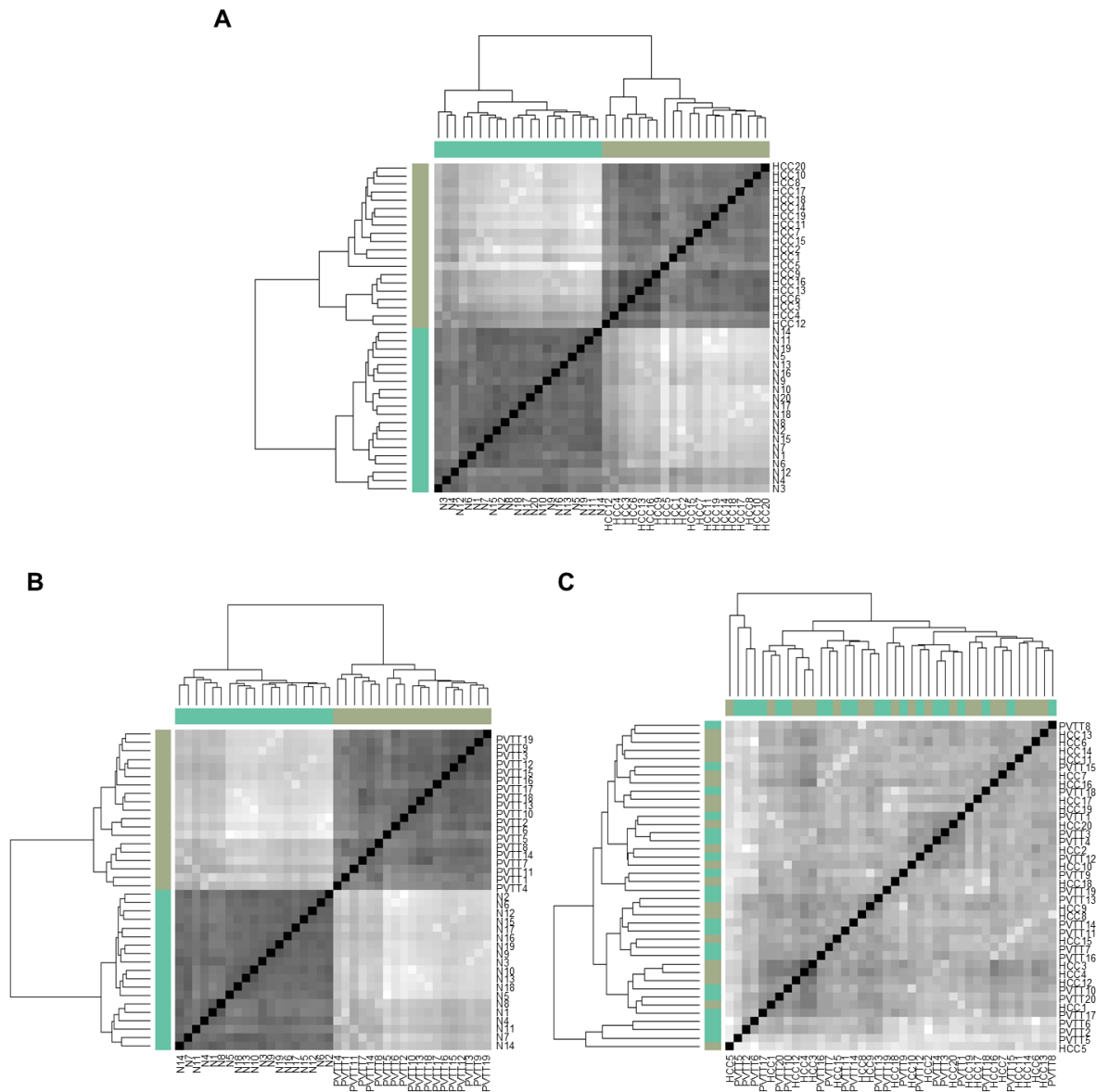

Supplement: Supplementary file 1 — Supplementary figure. [file jcav16p1754s1.pdf]
